# Antimicrobial Susceptibility of Clostridium Difficile Clinical Isolates in Iran

**DOI:** 10.5812/ircmj.5189

**Published:** 2013-08-05

**Authors:** Mehdi Goudarzi, Hossein Goudarzi, Masoud Alebouyeh, Masoumeh Azimi Rad, Farahnaz Sadat Shayegan Mehr, Mohammad Reza Zali, Mohammad Mehdi Aslani

**Affiliations:** 1 Research Center for Gastroenterology and Liver Diseases, Shahid Beheshti University of Medical Sciences, Tehran, IR Iran; 2 Department of Microbiology, Shahid Beheshti University of Medical Science, Tehran, IR Iran; 3 Department of Microbiology, Pasteur Institute, Tehran, IR Iran

**Keywords:** Clostridium Difficile, Antibiotic Resistance, Clindamycin

## Abstract

**Background:**

*Clostridium difficile* infection (CDI) is major growing problem in hospitals and its high incidence has been reported in recent years.

**Objectives:**

The aim of this study was to investigate the antimicrobial susceptibility patterns of *C. difficile* clinical isolates against antibiotics commonly used for treatment CDI in hospitalized patients.

**Material and Methods:**

During a 12 month study, 75 *C. difficile* isolates were collected from 390 patients with CDI. All samples were treated with alcohol and yeast extract broth. The treated suspensions were cultured on a selective cycloserine cefoxitin fructose agar (CCFA) supplemented with 5% sheep blood and incubated in anaerobic conditions, at 37 °C for 5 days. Cdd-3, tcdA and tcdB genes were identified using PCR assay.

**Results:**

The prevalence of A^+^B^+^ , A^+^ B^-^ and A^-^ B^+^ strains were 64(85.3%), 5(6.7%) and 6(8%) respectively. In vitro susceptibility of 75 clinical isolates of *C. difficile* to 5 antimicrobial agents, including metronidazole, vancomycin, clindamycin, erythromycin and cefotaxime were investigated by Clinical and Laboratory Standards Institute (CLSI) agar dilution method. Metronidazole and vancomycin had good activity against *C. difficile* isolates with MIC90s of 2 and 1 µg/ml, respectively. Seventy one (94.6%) of strains was inhibited by concentrations that did not exceed 2µg/ml for metronidazole. Resistant to metronidazole observed in 5.3% of isolates. Forty three (57.3%) of the isolates were resistant to erythromycin. Of 43 resistant strains to erythromycin, 9 (12%) isolates had high-level MIC of more than 64 µg/ml. All strains were resistant to cefotaxime. Sixty seven (89.3%) isolates were resistant to clindamycin (MIC90s > 256 µg/ml) and only 6.7% were sensitive to clindamycin. Multidrug-resistant (three or more antibiotics) was seen in 36(48%) isolates.

**Conclusions:**

Metronidazole and vancomycin still seem to be most effective drugs for treatment CDI.

## 1. Background

*Clostridium difficile* is gram-positive rod, spore forming, strict anaerobic bacillus and the major cause of nosocomial diarrhea (1). *C. difficile* is responsible for a spectrum of *C. difficile* infection (CDI) that can be ranged from mild, self-limiting diarrhoea to a severe colitis, pseudomembranous colitis or toxic megacolon (2). Toxins A and B from *C. difficile* are major factors which initiate the creation of CDI. Both of the toxins induce mucosal injury and colitis (3). CDI appears as a major complication of antibiotic therapy and is linked with hospital admission. Exposure to almost all classes of antibiotics has been associated with CDI (4, 5). In healthy persons, the growth of this bacterium is controlled by the intestinal normal flora. The use of broad-spectrum antimicrobials may cause depletion of the patient’s normal protective bowel microbiota and promote proliferation of toxigenic *C. difficile*. Therefore Antimicrobial therapy plays a central role in the development of CDI (4, 6).

Metronidazole and vancomycin are the first choice drugs for treatment of CDI but there is a high incidence of relapses (7). Several studies have proven that these two antibiotics are the mainstays for the treatment of mild to moderate disease (7, 8). Decreased susceptibility and increased resistance to metronidazole has caused to change standard antimicrobial therapy for CDI (9). Teicoplanin as a one of glycopeptide antibiotics have as equally effective as metronidazole and should be reserved for patients who cannot tolerate metronidazole (10). The effective treatment of CDI is considerably challenged with the emergence of new multi-drug resistant epidemic strains of these bacteria (11). In clinical laboratories, because of the need for anaerobic facility, expert technician and cost, antibiotic susceptibility testing is not routinely performed for *C. difficile*. Although in the most of studies susceptibility of *C. difficile* to metronidazole and vancomycin has been reported but recent studies described increase resistant and reduced sensitivity to metronidazole and vancomycin (12, 13). Information about CDI and also antimicrobial resistance profiles of *C. difficile* isolates in Iran is very sparse, but reports from Europe and North America indicates that prevalence of infections caused by *C. difficile* and resistance against antibiotics commonly used for treatment of this bacteria is increasing rapidly (14).

## 2. Objectives

The aim of this study was to investigate the antimicrobial susceptibility patterns of C.difficile clinical isolates against antibiotics commonly used for treatment CDI in hospitalized patients.

## 3. Material and Methods

### 3.1. Bacterial Isolates

A total of 75 clinical isolates of *C. difficile* were recovered from the 350 stool specimens of patients with CDI who were referred to the Research center for Gastroenterology and Liver Disease (RCGLD) as a referral laboratory during November 2010 to Oct 2011 were included in this study. A questionnaires containing different clinical and personal data i.e. clinical symptoms, antibiotic usages and underlying conditions was completed for all person. Diarrhea was defined as the passage more than 3 loose or watery stools during a 24-h period (15).

All the stool samples were transported to the laboratory and were processed immediately. Stool specimens were treated with alcohol and yeast extract broth. For alcohol treatment about 1 g of stool was mixed with an equal volume of 95% ethanol, slowly vortex and held at room temperature for 2 min (16). The treated suspensions were cultured on cycloserine- cefoxitin fructose agar (CCFA,) supplemented with 5% sheep blood for isolation of *C. difficile*. For yeast treatment, about 1 g of stool was mixed with an equal volume of yeast extract broth (Yeast extract granulated; Merck, Germany) and then treated suspensions were cultured on CCFA supplemented with 5% sheep blood. Plates were incubated in anaerobic conditions (Anoxomat: MART Microbiology B.V. the Netherlands, 0% O2, 10%H2, 10% CO2, 80% N2) at 37° C for 48 h. All plates were monitored daily up to 5days. Negative cultures were maintained in incubator up to 7 days. *C. difficile* isolates were presumptively identified by characteristic morphology of colony, specific horse-stable odor, Gram stain, green- chartreuse fluorescence under a ultraviolet (UV) light. Samples confirmed as a *C. difficile* were stored in cooked meat broth (Cooked Meat Medium: Himedia) at 4°C and were subjected to further molecular identification.

### 3.2. DNA Extraction and PCR of Toxigenic Genes

DNA was extracted from bacteria on CCFA medium by Using QIAamp DNA isolation columns (Qiagen, Hilden, Germany) according to the manufacturer’s procedure. The presence of PaLoc accessory gene cdd-3 was detected by PCR as described previously by Cohen et al. ( 17 ). All C.difficile isolates were subjected for the determination of toxin genes. The detection of Toxin A gene (tcdA) and toxin B gene (tcdB) was performed by the methods described by Cohen et al.( 17 ) Primer sequences used for detection of cdd-3, tcdA and tcdB genes and their fragment size are presented in Table 1. The PCR reactions for detection tcdA and tcdB genes were done a total volume of 25 µL The reaction mixture contained 1x buffer (10 mM Tris-HCl, 50 mM KCl), 1.5 mM MgCl2, 0.2µM of each deoxynucleoside triphosphate, 0.5µM of TA1 and TA2 primers, 0.5µM of TB1 and TB2 primers, and 1.5 U of Takara Taq (Takara Shuzo Co., Ltd., Shiga, Japan). 

**Table 1.** Primers Sequence Used for Amplification cdd3, tcdB and tcdA Genes

Gene	Primer	Nucleotide sequence	Fragment Length (bp)
**Cdd-3**	Tim6	5´ TCC AAT ATA ATA AAT TAG CAT TCC A 3´	622
	Struppi6	5´ GGC TAT TAC ACG TAA TCC AGA TA 3´	
**TcdA**	TA1	5´ATG ATA AGG CAA CTT CAG TGG 3´	624
	TA2	5´TAA GTT CCT CCT GCT CCA TCA A 3´	
**TcdB**	TB1	5´GAG CTG CTT CAA TTG GAG AGA 3´	412
	TB2	5´GTA ACC TAC TTT CAT AAC ACC AG 3´	

PCR conditions for amplification of 624 bp fragment of the tcdA gene was done by thermocycler (AG 22331; Eppendorf, Hamburg, Germany) as follows: initial denaturation at 5 min at 95ºC, followed by 35 cycles of 1min at 95 ºC, 1 min at 58 ºC, and 1.5 min at 72 ºC; and final extension at 72 ºC for 10 min to end amplification process. For amplification of 412 bp fragment of the tcdB gene the following time-temperature profile was used: 5 min at 94ºC for initial denaturation, 35 cycles of 1min at 94 ºC, 1 min at 51 ºC, and 80s at 72 ºC; and a final extension cycle of 5 min at 72 ºC. Amplified fragments were separated by 1.2% agarose gel electrophoresis at 80V for 2h. Finally, fragments were stained by ethidium bromide and detected under UV light.

### 3.3. Minimum Inhibitory Concentration (MIC)

The antimicrobial susceptibility profile for all isolates was determined by estimating MIC of the 5 antibiotics using agar dilution method according Clinical Laboratory and Standards Institute (CLSI; formerly National Committee for Clinical Laboratory Standards) criteria for anaerobes (M11-A6) (18). The MIC was defined as the lowest concentration of each antimicrobial agent that inhibited visible growth of the tested isolate. The following antimicrobial agents were used in this study: metronidazole, vancomycin, cefotaxime, clindamycin, erythromycin (Sigma-Aldrich, St. Louis, Mo). The ranges of MIC value used for antimicrobial agents were including: metronidazole 0.125 to 32 µg/ml; vancomycin 0.25 to 16 µg/ml; cefotaxime 4 to 512 µg/ml; clindamycin 0.5 to 256 and erythromycin 0.5 to 32 µg/ml. In brief, serial twofold dilutions of antibiotics were incorporate in to enriched Brucella agar (Oxide supplemented with 5% defibrinated sheep blood, 5 µg/ml haemin and µg/ml vitamin K1) for determination of antibiotic susceptibility. The stock solutions of each drug were prepared in accordance with manufacturer’s instructions and kept at –20°C. From these stock solutions, working solutions were made in distilled water to be incorporated into the Brucella blood agar media. Media with different concentrations of each antibiotic were prepared by adding defined amount of each antibiotic to cooled Brucella agar media. The bacterial suspension obtained from overnight cultures. The turbidity of each bacterial suspension was adjusted equivalent to a no. 1.0 McFarland standard and 20 µl of them were inoculated on Brucella agar plates containing different concentrations of each antibiotic and plates without antibiotics as control. Control plates were included with each test run of susceptibility testing. Antibiotic resistance was defined as follows: MIC ≥ 32 µg/ml for metronidazole, MIC ≥ 64 µg/ml for cefotaxime, MIC ≥8 µg/ml for clindamycin, MIC ≥ 8 mg/L for erythromycin according to the Clinical and Laboratory Standard Institute (CLSI) recommendations (18).

## 4. Results

**Figure 1.**
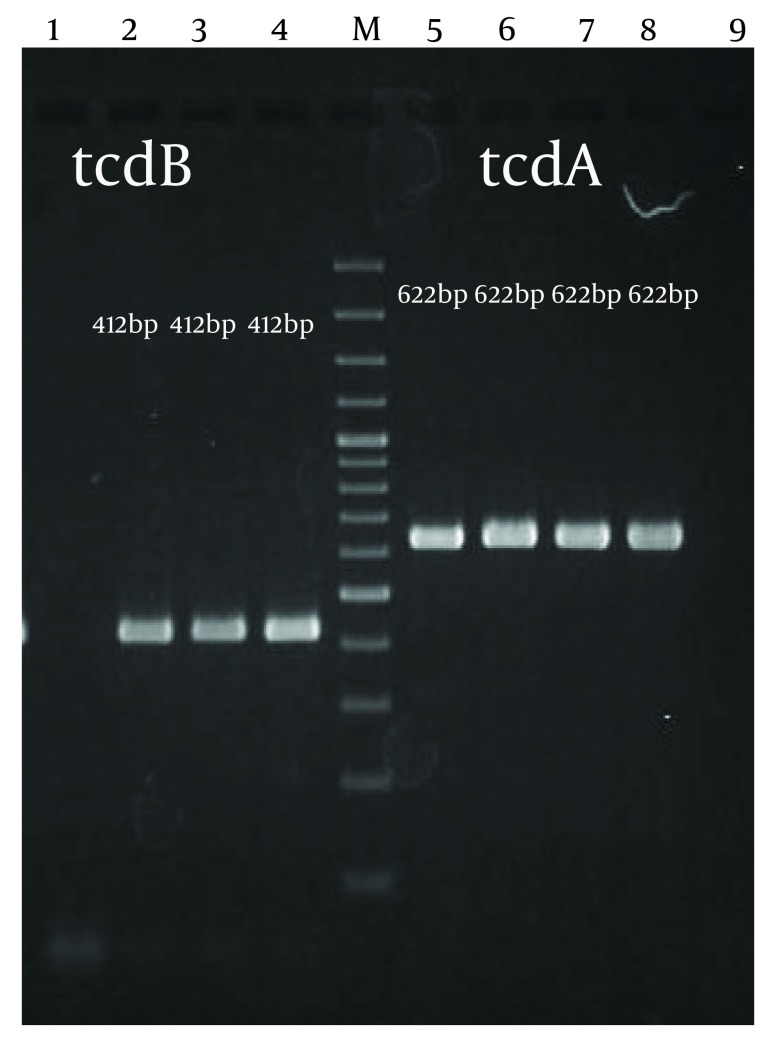
Detection of tcdA and tcdB genes by PCR.lane 1 negative control of tcdB gene, lan2 and 3 PCR tcdB gene,lan 4 control positive of tcdB gene, lane 5,6 and 7 PCR tcdA gene, lane 8 control positive of tcdA gene, lane 9 negative control of tcdA gene, lane M, DNA ladder, 100 bp

Clinical features of 75 patients with CDI are given in Table 2. The patients were distributed in 7 hospital department. All patients hospitalized in gastroenterology ward had history of previous surgery and use of proton pomp inhibitors. The most common underlying disease was renal failure, infectious disease and cancer. Ninety two percent of patients had antibiotic therapy. Over 50% of them were treated with the Beta-lactam antibiotic. Only 6 patients (8%) did not receive a specific treatment. The toxin profiles A ^+ ^B ^+ ^, A ^+ ^B ^- ^and A ^- ^B ^+ ^accounted for 64(85.3%), 5(6.7%) and 6(8%) of studied strains respectively. PCR products of the tcdA and tcdB genes are shown in Figure 1. All patients hospitalized in ICU and oncology ward were (A ^+ ^B ^+ ^) and had history of usage of antibiotics such as beta lactams, aminoglycosides and flouroquinolones. A total of 6 (A ^− ^/B ^+ ^) strains were isolated from different wards including 3 from ICU, 2 from infectious and 1 from oncology ward. All patients with profile A ^− ^/B ^+ ^had underlying morbidity, leucosytisis and history of previous use of cephalosporines, aminoglycosides and Beta-lactams antibiotics. Surprisingly, 5 strains were toxin A positive but toxin B negative. They were isolated from patients with fever, abdominal pain and previous use of antibiotics. 

**Table2.** Demographic and Epidemiological Characteristics of 75 Patients with CDI

Characteristic	No. (%)
**Gender**	
Male	39 (52)
Female	36 (48)
**Age, years**	
≤50	41 (54.6)
51–65	11 (14.7)
> 65	23 (30.7)
**Laboratory parameters**	
Nuetropenia	7 (9.3)
Leucocytosis	19 (25.3)
blood in stool	12 (16)
**Clinical parameters**	
Fever	36 (48)
Abdominal pain	51 (68)
**Duration of diarrhea(more than twice per day: days)**	4.9 ± 5.8
**Duration of hospitalization**	10.5
**Abdominal surgery**	35 (46.6)
**Previous surgery**	17 (22.7)
**Previous hospitalization**	38 (50.7)
**Use of immunosuppressive drugs**	12 (16)
**Use of protone pump inhibitors**	19 (25.3)
**Nasogastric tube**	10 (13.3)
**Chemotherapy**	2 (2.7)
**Diagnosis on admission**	
Hematological malignancies	5 (6.7)
Cancer	7 (9.3)
Renal failure	11 (14.6)
Infectious disease	5 (6.7)
Chronic disease	4 (5.3)
others	43 (57.3)
**Hospital ward**	
Internal medicine	20 (26.7)
Intensive care unit	15 (20)
Infectious ward	14 (18.7)
Surgical ward	10 (13.3)
Oncology	10 (13.3)
Gastroenterology	4 (5.3)
Pediatric	2 (2.7)
**Previous use of antibiotics**	
Penicillin	47 (62.6)
cephalosporin	41 (54.6)
clindamycin,	21 (28)
aminoglycoside	14 (18.6)
fluoroquinolone	11 (14.6)
metronidazole	8 (10.6)
vancomycin	4 (5.3)
other	15 (20)

In vitro susceptibility of the *C. difficile *isolates to 5 antibiotics tested and the range of Minimum Inhibitory Concentration required to inhibit the growth of 50% of organisms (MIC _50 _) and Minimum Inhibitory Concentration required to inhibit the growth of 90% of organisms (MIC _90 _) are summarized in Table 3. All isolates were resistant to cefotaxime. Of all the isolates resistant to cefotaxime , 27 (36%) of isolates had MIC ≥ 64 µg/ml, 40 (53.3%) had MIC ≥ 128 µg/ml, 5 (6.7%) had MIC ≥ 256 µg/ml and 3(4%) had MIC ≥ 512 µg/ml. Increased resistance to metronidazole was observed for 4 (5.3%) of isolates (three strains for which the MICs were 32µg/ml, the remaining one strain for which the MIC was 64 µg/ml). The MIC values of metronidazole for remaining 71 (94.7%) of isolates was ranged from 0.125 to 2 µg/ml. 

**Table 3.** Antimicrobial Susceptibilities of 75 Clostridium Difficile Isolates to 5 Antimicrobial Agents

agent	MIC(µg/ml)			No.(%)of isolates			MICI nterpretive Breakpoints (S/I/R) ^a^ (S/I/R)
	Range	50%	90%	S	I	R	
**Metronidazole**	0.125-32	0.5	2	71 (94.7)	0 (0)	4 (5.3)	≤ 8/16/32 ≥
**Vancomycin ** ^b^	0.25-16	1	1	92	0 (0)	6 (8)	≥ 2
**Cefotaxime**	4-512	> 128	256	0 (0)	0 (0)	75 (100)	≤ 16/32/64 ≥
**Clindamycin**	0.5-256	32	256	5 (6.7)	3 (4)	67 (89.3)	≤ 2/4/8 ≥
**Erythromycin**	0.5-32	4	> 32	23 (30.7)	9 (12)	43 (57.3)	≤ 2/4/8 ≥

^a^MIC breakpoints applied were those recommended for anaerobes by the Clinical and Laboratory Standards Institute (CLSI)

^b^Vancomycin MIC breakpoint was recommended by the European Committee on Antimicrobial Susceptibility Testing (www.eucast.org)

The results of metronidazole MIC were as fallow: 5 (6.7%) of isolates had MIC 0.125 µg/ml, 14 (18.7%) had MIC 0.25 µg/ml, 37 (49.3%) had MIC 0. 5 µg/ml, 12 (16%) had MIC 1 µg/ml, 3 (4%) had MIC 2 µg/ml, 3 (4%) had MIC 32 µg/ml and 1(1.3%) had MIC 64 µg/ml. There was any intermediate isolate for metronidazole. Among metronidazole resistant isolates, one strain with MIC ≥ 64 µg/ml was isolated from a 66-years-old HIV positive patient who had undergone gastrointestinal disease. The other patient infected by metronidazole resistant strain was a child with malignancy who had been received metronidazole treatment. As it was shown, 43(57.3%) and 67(89.3%) of the isolates were resistant to erythromycin and clindamycin respectively. Out of 43 resistance isolates to erythromycin, 13 (17.3%) of isolates had MIC ≥ 8 µg/ml, 12(16%) had MIC ≥ 16 µg/ml, 9 (12%) had MIC ≥ 32 µg/ml and 9 (12%) exhibited MIC ≥ 64 µg/ml. Nine (12%) of isolates were intermediate to erythromycin. From 67 resistance isolates to Clindamycin, 7 ( 9.3% ) of isolates had MIC ≥ 8 µg/ml , 10 ( 13.3% ) had MIC ≥ 16 µg/ml, 13( 17.3%) had MIC ≥ 32 µg/ml, 29 (38.6%) had MIC ≥ 128 µg/ml and 8 (10.6%) had MIC ≥ 256 µg/ml. Just 3(4%) of all isolates were intermediate to clindamycin.

All of *C. difficile* strains except six of them were inhibited by vancomycin at MIC ≤ 2 µg/ml. Out of 6 resistance isolates to vancomycin, 4(66.7%) of isolates had MIC 2 µg/ml and 2 (33.3%) had MIC 4 µg/ml. Two isolates with high value MIC to vancomycin (MIC 4 µg/ml) were positive for both toxin A and B (A^+^ B^+^) and recovered from the same hospital. The MIC90s of clindamycin and cefotaxim were alike (256µg/ml). All of *C. difficile* strains were inhibited by vancomycin at similar MIC_50_ and MIC_90_ 1 µg/ml. In this study metronidazole and vancomycin showed good in vitro activity against all strain tested, with MIC_90_ of 1 and 2 µg/ml respectively. According to our results highest (100%) and lowest (5.3%) levels of resistance were related to cefotaxim and metronidazole respectively.

Multidrug-resistant (MDR) was defined as resistance to at least three or more antibiotics.15 Of 75 isolates tested 36(48%) were MDR. In particular, thirty nine (52%) of isolates were resistant to at least two drugs, Thirty one (41.3%) of isolates were resistant to at least three drugs and 5(6.7%) of the isolates were resistant to four drugs. Frequencies of resistance to two or more antibiotics are summarized in Table 4. MDR strains to three or more tested antibiotics were isolates from hospitalized patients in ICU, internal medicine, infectious, oncology and gastroenterology wards respectively. The predominant resistance profile among our isolates were included resistance to 2 antibiotices (cefotaxim, clindamycin) and 3 antibiotices (cefotaxim, clindamycin, erythromycin), which were common among 31(41.3%) and 30 (40%) isolates. 

**Table 4.** Distribution of Antibiotic Resistance Profile Among *C. difficile *Strains

Resistance group	Toxin A positive	Toxin B positive	Toxin A and B positive	Total, No.
**CEF,CLI,ERY,MTZ**	-	1	2	3
**CEF,CLI,ERY,VAN**	-	-	2	2
**CEF,CLI,ERY**	3	2	25	30
**CEF,CLI,VAN**	1	-	-	1
**CEF,CLI**	1	3	27	31
**CEF,ERY**	-	-	7	7
**CEF, MTZ**	-	-	1	1

## 5. Discussion

CDI is a potentially fatal illness with an increasing incidence worldwide and responsible for 10-20% cases of antibiotic-associated diarrhea (AAD) and almost all cases of colitis associated with antibiotic therapy (6, 19, 20). In this study we studied susceptibility pattern of the 75 clinical isolates of *C. difficile* to 5 different antibiotics as common therapeutic drugs in hospitalized patients. Of the total 75 isolates, 6 (8%) were A^-^B^+^ strains. Several investigators believe that CDI caused by A^-^B+ strains is increasing (21). The prevalence of A^-^B^+^ strains varies depending on geographic region and country studied. In Europe, 6.2% of *C. difficile* isolates were A^−^B^+^ variant (22). In a study conducted in Canada the prevalence of A^-^B^+^ strains was 2.3% (23). In Shanghai 33.3% of the isolated strains were A^-^B^+^ strains while in Stockholm did not identify any A^-^B^+^ strain (24). In Korea, A^−^B^+^ variant was 25.7% of *C. difficile* isolates in 2010 (25). The prevalence of A^-^B^+^ strains in Iran was much lower than Korea and Shanghai.

The MIC values for metronidazole have been reported differently by several researchers. In 2002, the MIC_50_ and MIC_90_ of metronidazole at 50% of isolates tested were 0.5 and 4 µg/ml, respectively in Spain (26). Lamothe et al. showed that all strains were susceptible to metronidazole and inhibited by MIC_50_ and MIC_90_ that did not exceed 0.25µg/ml and 0.5µg/ml respectively (27). In a study conducted in Sweden, the MIC of metronidazole for 238 *C. difficile* isolates ranged from 0.032 to 1 µg/ml (28). Poxton et al. showed that resistance to antibiotics during the three periods of the study has changed and also reported that the MIC_50_ and MIC_90_ values of metronidazole for 179 isolates were 0.5 µg/ml (29). In 2008 in the United Kingdom, the MIC_50_ and MIC_90_ results of the metronidazole in 677 clinical isolates of *C. difficile* were 0.38 µg/ml and 1.0 µg/ml respectively (30). In a study done in Taiwan in 2011, Chien Ko et al. showed that all of strains were susceptible to metronidazole and the rate of MIC_50_ and MIC_90_ for metronidazole were 0.5 µg/ml and 1 µg/ml respectively (31).The data from present study showed that 71 (94.7%) of isolates were inhibited by 2 µg/ml of metronidazole and only 4 (5.3%) were resistance to metronidazole. Resistance to metronidazole in different countries is gradually increasing. Also isolates with decrease susceptibility to metronidazole has been confirmed by several investigators. Pelaez et al. reported the increased rate of resistant to metronidazole from 6.3% to 12% during three years (13, 26). Wong et al. showed that from 100 *C. difficile* isolates only single isolate was resistant to metronidazole and had MIC of 64 µg/ml (32).In compare to studies were performed in Taiwan (31), Canada (27), Sweden (24) and UK (30) , a high resistance to metronidazole were seen in our study. This high resistance to metronidazole in Iran can be caused by indiscriminate use of metronidazole in the treatment of CDI and altered in the ability of bacteria to activate the drug (33). Although resistant to metronidazole is gradually increasing but it is still an effective drug for treatment C. difficile-associated diseases (34).

The data from our investigation show that 90% of isolates were inhibited by 1 µg/ml of vancomycin. Sixty nine (92%) of isolates were inhibited by concentration that did not exceed 2 µg/ml. Decreased susceptibility to vancomycin in *C. difficile* isolates has been reported previously (35). The study was conducted in 2004-2006 in Poland, has shown that all isolates were inhibited by concentrations that did not exceed 2 mg/ml for vancomycin (5). Another study that were done on United States, South America, and Europe isolates showed that all isolate except one were inhibited by vancomycin at a concentration of 2.0 µg/ml (36). In Spain, decreased susceptibility to vancomycin reported in 10% of clinical strains of *C. difficile* (26). In another study that was conducted in Canada showed that all isolates were susceptible to vancomycin (27). Our finding about vancomycin is in accordance with recent data. Although decreased susceptibility to vancomycin among *C. difficile* isolates has been reported but it is still used as effective drug for CDI treatment.

All 75 isolates were resistant to cefotaxime with MIC90 more than 256 µg/ml .This data is consistent with some earlier reports (27, 37). In 2005, brazier et al. showed that all 271 C. difficile isolates were resistance to cefotaxim with MIC ≥ 64µg/ml (38). In 2006, Lamothe et al. reported that all *C. difficile* isolates were resistance to cefotaxim with MIC50 and MIC_90_ ≥ 128 µg/ml (27). In another study conducted in Kuwait in 2002, all studied strains were resistance to cefotaxim with MIC50 and MIC_90_ 96 and ≥ 256 µg/ml respectively (37). Resistance to cefotaxim among our isolates may be related to improper usage of this antibiotic for treatment of other infections, increase use of other beta lactam antibiotics in hospital and acquisition of resistant during hospitalization.

In spite of limitation in the use of clindamycin due to its association with the induction of C. difficile diarrhea and a high risk of inducing CDI but resistant to clindamycin have been widely reported (39). The MIC of clindamycin for our isolates ranged from 0.5 to 256 µg/ml. The clindamycin exhibited higher MIC than other antimicrobial agents tested with MIC_90_ of more than 256µg/ml and had poor activity against the isolates. The resistance rate to clindamycin was 89.3% in our study, which was lower than Canada (90.9%), but was higher than those in Korea (60%), China (81.3%), Kuwait (48%), Sweden (65%) and Taiwan (46%) (23-25, 31, 37, 40). The reason for resistance to clindamycin could be mediated resistance to other macrolide and also their widespread use in the hospital and the community.

The resistance rate to erythromycin was 57.3% in our study, which was lower than those in China (85.3%), Scotland (94.8%) and was higher than those in Germany (49 %), Hungary (25%) and Sweden (13.8%) (40, 41). The possible reasons of high resistant rate to erythromycin in present study may be related to use of erythromycin in treatment of disease caused by *C. difficile* and common infections, increase exposure of this isolates to new macrolide, efflux of the drug and ribosomal methylation (42). Cross resistance between clindamycin and macrolides is well described by several investigators (42). In this study 30 isolates were simultaneously resistant to both clindamycin and erythromycin antibiotics. Cross resistance to clindamycin and erythromycin is most likely due to cross resistance with other macrolide, lincosamide antibiotics and the presence of erythromycin ribosomal methylase B (ermB) genes and also acquired resistance genes via a non-plasmid-mediated mechanism (22).

Other studies showed that percentage of *C. difficile* multidrug-resistant strains varies from one geographic region to another and ranges between 2.5% and 66 % (24, 25). Our study showed that, 48% of isolates were MDR. According to study that was conducted by The European Study Group on *Clostridium difficile* (ESGCD) in 2008, 25.9% of isolates were resistance to at least three antibiotics (14).The frequency of MDR among isolates of *C. difficile* is increasing. The studies were performed by several investigators exhibited that resistance to erythromycin, clindamycin and moxifloxacin increased among *C. difficile* isolates (29). A high incidence of MDR strains was found in ICU and internal medicine wards in our study. It could be attributable to high usage of antimicrobials agents in ICU. Continued use of antibiotic for treatment of CDI should be supported by monitoring of antimicrobial susceptibility to prevent the spread of resistant isolates and also eliminate the need of antibiotics for a prolonged period (43).

In conclusion, this study has shown that resistance to metronidazole and vancomycin among our isolates was very low while full resistant to cephalosporines among our *C. difficile* isolates was common. Although resistant to metronidazole has seen among our isolates but it seems that metronidazole and vancomycin can be effective drugs for treatment of CDI. According to our findings, cefotaxim, clindamycin, erythromycin are not effective drugs for treatment of CDI. Progressive increase in resistant to cefotaxim, clindamycin, erythromycin and multiple resistances to antibiotics in present study, may be related to increased usage of these antibiotics for treatment of CDI and ability of strains in acquisition of resistance genes. Continuous Surveillance for *C. difficile* multidrug-resistant strains is necessary to prevent the further spread of resistant isolates.
